# Evaluating the Efficacy, Tolerability, and Outcomes of Topical Tripeptide/Hexapeptide Formulations Before and After Liposuction of the Medial Thighs

**DOI:** 10.1093/asjof/ojaa055

**Published:** 2020-12-30

**Authors:** Brannon Claytor, Laurie Casas, Mary Ziegler, Alan D Widgerow, Michaela Bell

## Abstract

Regenerating Skin Nectar with TriHex Technology (RSN) has been shown clinically to promote healing and outcomes post procedures. TransFORM Body Treatment with TriHex Technology (TFB) has demonstrated clinically to improve lipid droplet dissolution and patient-reported outcomes post procedure. Histologically, both have been proven to regenerate collagen and elastin. The objective is to evaluate postprocedural recovery, histological, and gene expression changes of medial thigh liposuction in participants using RSN and TFB on one thigh in comparison with a bland moisturizer on the other. Participants were randomized to apply RSN pre and post procedure and TFB post procedure to one thigh and a bland moisturizer to the other. After topical pretreatment, participants underwent bilateral medial thigh liposuction. Assessments included induration measurements, ultrasounds, blinded investigator assessments, participant assessments, photography, and biopsies for 10 weeks post procedure. Blinded investigator assessments of induration, edema, and subcutaneous fibrous banding had less severity at weeks 1, 2, and 4 on the RSN/TFB thigh, corresponding with induration measurements. Ultrasound images showed less fluid infiltration, edema, and induration on the RSN/TFB side over the bland moisturizer at 2 weeks. Gene expression confirmed a hastened inflammatory phase converting more rapidly to the anti-inflammatory regenerative healing environment with evidence of extracellular remodeling only present on the RSN/TFB side at week 4, and histological biopsies demonstrated improved collagenesis and elastogenesis. RSN used before surgical procedures combined with RSN and TFB post procedure has demonstrated remodeling of the extracellular matrix, accelerating healing, and initiation of anti-inflammatory genes.

**Level of Evidence: 4:**

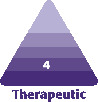

Surgical aesthetic procedures are on the increase with a trend in body shaping procedures.^1^ Surgical outcomes and recovery time are paramount for physicians and patients. Topically preparing the skin before surgical procedures has become a recognized concomitant treatment, in addition, used post procedurally to aid in healing and outcomes. Regenerating Skin Nectar with TriHex Technology (RSN; Alastin Skincare, Inc., Carlsbad, CA) is an anhydrous gel with a proprietary recycling mechanism to aid in the removal of aged, damaged fragments and restore an optimal environment with new, healthy collagen and elastin.^2^ It is the combination of synergistic, targeted peptides and active ingredients that have been shown to promote healing and outcomes following laser treatments as well as surgery along with histological evidence that confirms remodeling of the extracellular matrix (ECM).^3^

TransFORM Body Treatment with TriHex Technology (TFB; Alastin Skincare, Inc., Carlsbad, CA) has also shown histologic evidence of regenerated collagen and elastin and, in addition, improves lipid droplet dissolution. Facilitated through a liposome delivery system, the actives are deposited into the base of the hair follicle where this reservoir continually delivers the product to the dermal white and then subcutaneous white adipose tissue. Hexapeptide-11 has been proven to accelerate (upregulate) the process of autophagy, encouraging lipid droplet breakdown. In vitro modeling shows upregulated macrophage recruitment to damaged fat cells with in vivo trials confirming increased and hastened fat volume reduction.^4-6^ In a recent study, patients using TFB had a noticeable reduction in postprocedural soft tissue changes and improved patient-reported recovery outcomes compared with patients not using the topical.^7^

This split body, randomized, blinded study was designed to incorporate the preprocedural topical application of RSN and postprocedural topical application of RSN and TFB to determine gene expression changes, histological alterations, recovery, and outcomes. Liposuction of the medial thighs was determined to be the most advantageous, thus avoiding crossover contamination of the 2 sides. In addition, the manipulation of adipose tissue with lipid droplet breakdown products likely caused by liposuction made this a suitable model for testing skin changes subjectively and objectively in this patient group.

## METHODS

Six female participants were enrolled in this Institutional Review Board-approved (IntegReview, Austin, TX), split leg, randomized, double-blind clinical study. The study was conducted over a period of 8 ½ months from August 8, 2019, to March 17, 2020, and enrollment was for 5 months between August 8, 2019, and December 23, 2019. The study was conducted in accordance with the guidelines set forth in the Declaration of Helsinki. Eligible participants were men and women, 18 years of age or older, electing medial thigh liposuction, and willing to apply 2 topicals before and after the procedure to the procedural area. Participants who were not good candidates for medial thigh liposuction, as determined by the physician, were excluded from participating. Pregnant or lactating participants were also excluded as well as participants planning on becoming pregnant during the study duration. Only participants who met the criteria to enroll were consented and assigned a number, in chronological order, and a corresponding blinded kit that contained RSN and Cetaphil Lotion (Galderma, Fort Worth, TX)-bland moisturizer. In each randomized kit, there were 2 blinded bottles exactly alike that were labeled right or left leg. At visit 1, the participant was instructed to use the assigned designated product to the medial thigh twice daily, for approximately 2–3 weeks, before visit 2 liposuction procedure. Procedures at visit 1 consisted of ultrasound scans, photography, skinfibrometer measurements, and biopsies, for participants who elected. At visit 2, participants had ultrasound scans, photography, and skinfibrometer measurements before the liposuction procedure. After the procedure, they were given additional blinded products (RSN, TFB, and 2 bland moisturizer bottles). Participants were instructed to continue to apply the 2 topicals given at visit 1 along with the additional products to each designated leg twice daily for the remainder of the 10-week follow-up. At every visit, participants were queried on product usage and dispensed additional products when needed. Postprocedure visits included weeks 1, 2, 4, 7, and 10. At each follow-up visit, procedures consisted of photography, ultrasound scans, skinfibrometer measurements, blinded investigator assessments, participant assessments, and biopsies at specified time points for those who elected. 

### Liposuction Procedure

The liposuction ports were identical in each case. Two incisions on each leg: the superior incision along the bikini line in the upper medial groin and the second along the medial aspect of the thigh 10 cm superior to the medial condyle of the knee. The liposuction volume was consistent at 250 mL per medial thigh with similar suction techniques. All participants received compression garments and were instructed to remove twice daily to apply topicals to the designated leg, allowing for the products to absorb and then reapply garment.

### SkinFibroMeter Measurements

The SkinFibroMeter (Delfin Technologies, USA, Miami, FL) consists of a 1.25-mm length indenter and 2 force sensors. The device is briefly pressed against the skin and the contact force is registered. The indenter imposes a constant deformation when the instrument is in full contact with the skin. The skin and the underlying upper subcutis resist the deformation, and the induration value in Newtons (N) is determined.

Measurements were taken at every visit and pre procedure at visit 2. The skinfibrometer was placed at 8, 10, and 12 cm from the top of the medial thigh on each leg, remote from the biopsy sites. Three measurements were recorded at each location.

### Biopsies

Of the 6 participants enrolled in the study, 5 consented to having punch biopsies, ages 18–43, and mean age 34. A 3-mm punch biopsy was performed at 3 visits on the right and left legs (visit 1 pretopical application and 2- and 4-weeks post liposuction). All punch biopsies were completed in the same location, with sites remote from the liposuction ports. The first biopsy was 8 cm inferior to the adductor insertion, the second biopsy was 10 cm, and the third was 12 cm inferior, along a line drawn from the insertion of the *adductor brevis* on the inferior ramus of the pubis to the medial condyle of the femur at the knee. The first 3 participants’ biopsy sets were sent to Genemarkers (Kalamazoo, MI) for gene expression analysis. Paired *t*-tests (*N* = 3, *P* < 0.05) were performed using TIBCO Spotfire software (Palo Alto, CA) for the gene expression analyses. The other 2 participants’ biopsies were sent to Laboratory and Pathology Diagnostics (Naperville, IL) for dermatopathology analyses.

### Ultrasounds

Using the Episcan I-200 with a 20-MHz probe (Longport, Inc., USA, Chadds Ford, PA), ultrasound images were taken on each leg at 3 measured locations (8, 10, and 12 cm from the top of the medial thigh), remote from the biopsy sites. Images were captured at 5-, 10-, 15-, and 20-mm depths at baseline, pretopical application, pre procedure at visit 2, and at every follow-up visit. To measure the transition/difference from the dermal to subcutaneous tissue, the difference between the dermal and subcutaneous intensity analysis was calculated. RDIS is relative dispersion, a measure of relative variation. A higher value means a more echogenic or brighter image, which in turn is indicative of denser tissue. Induration is represented by disruption of the subcutaneous dermal interface with infiltration of fluid from the subcutaneous region to the dermal segment, thus disrupting the compact nature of the dermis resulting in a less dense dermis.

### Investigator Assessments

At weeks 1, 2, 4, 7, and 10 post liposuction to the medial thighs, the blinded investigator assessed each leg for induration, edema, skin discoloration, ecchymosis, subcutaneous banding, and pain. Each assessment was made using a 0–4-point scale (0 = none, 1 = barely perceptible, 2 = slight, 3 = moderate, and 4 = severe). The Visual Analogue Scale (VAS) pain scale was also used to assess pain (0-10 scale).

### Participant Assessments

At every visit following the liposuction procedure, each participant was given a paper questionnaire by the study coordinator to assess each leg on a 0–3-point scale (0 = none, 1 = mild, 2 = moderate, and 3 = severe). The questionnaire utilized in the study is available in the Appendix. 

## RESULTS

Six female participants, ages 18–58, mean age 46, enrolled and completed the 10-week follow-up. Participants able to return to the office for a long-term follow-up were completed 10–11 months post procedure.

### Blinded Investigator Assessments

**Figure 1.**
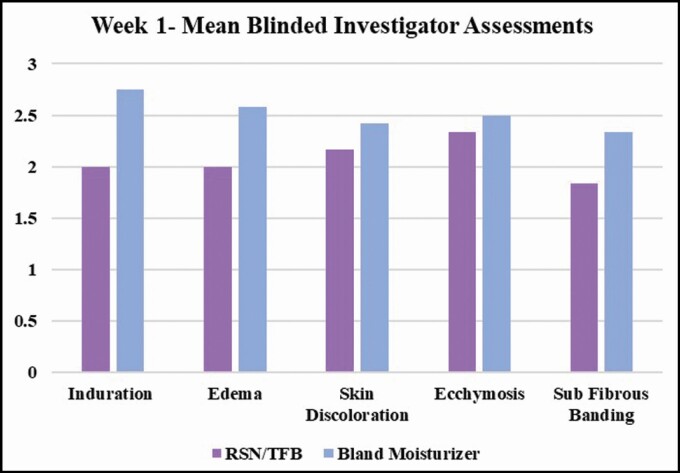
Mean blinded investigator assessments: at week 1, all healing parameters were graded less severe on the side using RSN/TFB. TFB, TransFORM Body Treatment with TriHex Technology; RSN, Regenerating Skin Nectar with TriHex Technology.

**Figure 2.**
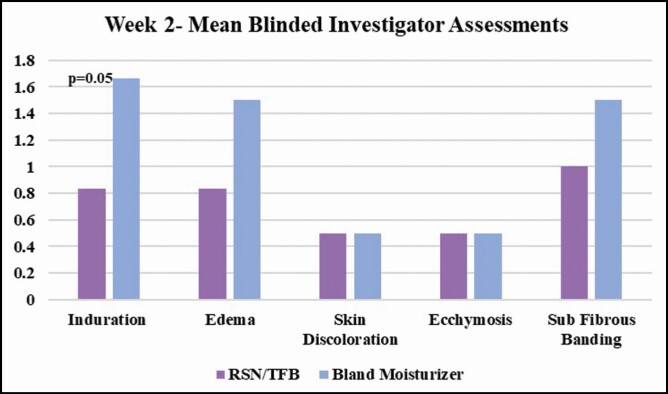
Mean blinded investigator assessments: at week 2, there was a statistically significant difference in induration between each leg, 0.833 for the topical treatment leg and 1.66 for the bland moisturizer leg (*P* = 0.05). Edema was also on trend with 0.833 for the topical treatment leg and 1.5 for the bland moisturizer leg. Subcutaneous banding was 1.0 for the topical treatment side and 1.5 for the bland moisturizer leg.

**Figure 3.**
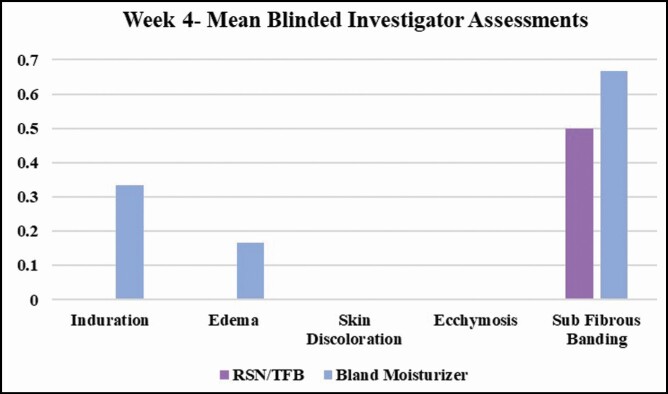
Mean blinded investigator assessments: at week 4, only mild subcutaneous banding was present on the topical treatment side (0.5), while on the bland moisturizer side, induration (0.33), edema (0.16), and subcutaneous banding (0.7) persisted.

Post liposuction, the mean blinded investigator assessments of induration, edema, and subcutaneous fibrous banding had less severity at weeks 1, 2, and 4 on the leg using the RSN and TFB (topical treatment). At week 1, skin discoloration, ecchymosis, and pain on the VAS were also less on the side using the topical treatment. At week 2, there was a statistically significant difference in induration between each leg, 0.833 for the topical treatment leg and 1.66 on the bland moisturizer leg (*P* = 0.05). Edema was also on trend with 0.833 for the topical treatment leg and 1.5 for the bland moisturizer leg. Subcutaneous banding was 1.0 for the topical treatment side and 1.5 for the bland moisturizer leg. At week 4, only mild subcutaneous banding was present on the topical treatment side 0.5, while on the bland moisturizer side, induration 0.33, edema 0.16, and subcutaneous banding 0.7 were present. At week 7, induration persisted on some of the participants’ leg using bland moisturizer. Edema increased slightly on both sides, which is typically seen clinically post liposuction, due to lifestyle. By week 10, all postprocedural healing assessments had resolved on the side using the topical treatments, while induration and subcutaneous banding were still present in some of the participants’ side using bland moisturizer. All data are tabulated and shown in **Figures 1-3**.

### Skinfibrometer Measurements

**Figure 4.**
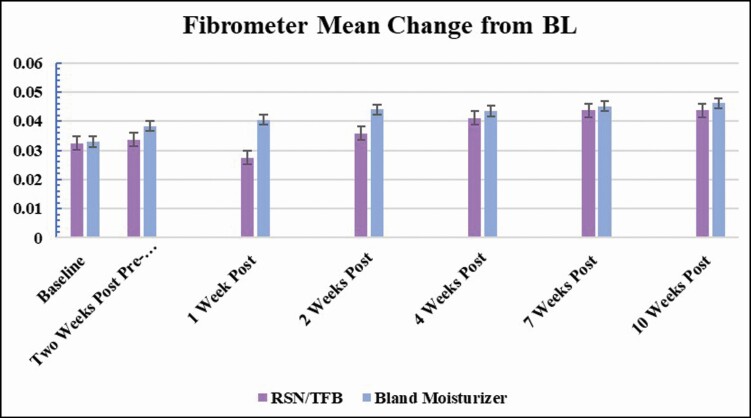
Skinfibrometer induration measurements: 3 measurements were taken at each of the 3 measured locations on both medial thighs. An increase from baseline indicates an increase in induration. Measurements were taken at every visit. The mean change from baseline was calculated for each leg at every visit. Weeks 1 and 2 post procedure showed the greatest difference in results with the side having the topical treatment of RSN and TFB showing the least change from baseline, whereas the leg using bland moisturizer had a greater increase from baseline indicating an increase in induration.

The mean change was calculated for each leg at every visit. Again, weeks 1 and 2 post procedure showed the greatest difference in results with the side having the topical treatment of RSN and TFB showing the least change from baseline, whereas the leg using bland moisturizer had a greater increase from baseline indicating an increase in induration. Although not statistically significant, this trend is maintained throughout the first 4 weeks (Figure 4).

### Ultrasound Images

Ultrasounds were taken at every visit on each leg at the same distance (8, 10, and 12 cm from the top of the medial thigh) and were analyzed by a blinded independent evaluator from Longport, Inc. Clinically, the major differences in induration were noted at 2 weeks; therefore, ultrasound analysis was focused at this time period. To measure the transition from the dermal to subcutaneous tissue, the difference between the dermal and subcutaneous intensity analysis was calculated. RDIS is relative dispersion, a measure of relative variation. A higher value means more echogenic or a brighter image, which in turn is indicative of denser tissue. Induration is represented by disruption of the subcutaneous dermal interface with infiltration of fluid from subcutaneous region to dermal segment, thus disrupting the compact nature of the dermis resulting in a less dense dermis.

**Figure 5.**
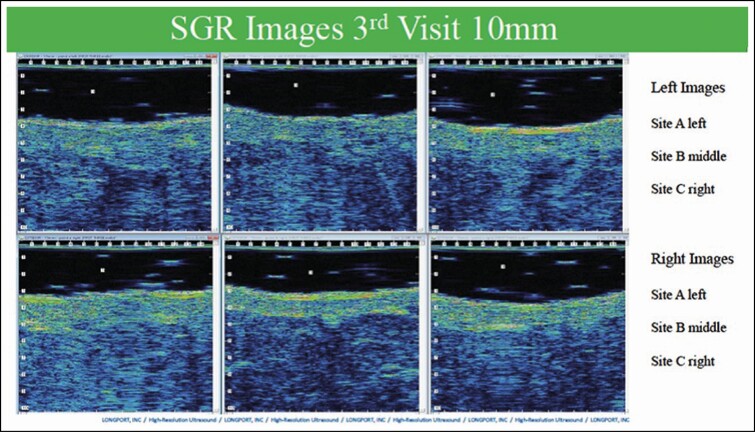
Ultrasound image: bland moisturizer on the top (Left Images) and RSN/TFB on the bottom (Right Images). Percentage change from the dermis to SUBQ L-AVG 127.60 R-AVG 155.1. The dermal image analysis indicates that the right dermis is more echogenic or brighter, suggesting it is denser. Combining the data analysis, the difference is 22%. A typical example of a comparison of fluid disruption into dermal segment representing induration and edema showing less disruption of dermal subcutaneous boundary on RSN/TFB side. TFB, TransFORM Body Treatment with TriHex Technology; RSN, Regenerating Skin Nectar with TriHex Technology.

Of the 6 participants, 5 showed an increased average density over 3 areas (less fluid infiltration, edema, and induration) on the RSN/TFB side over the comparator at the 2-week time point. Even in the 1 participant who did not show the overall advantage on the RSN/TFB side, the ultrasound in fact showed less induration in the middle image on the RSN/TFB side. Put another way, out of the 18 comparison points, the leg using RSN and TFB proved to have less induration in 15 of the 18 images (83.3%). In addition, if just examining the middle area, being representative of the most consistent area for liposuction, all 6 participants showed improved results on the side using RSN and TFB (Figure 5).

#### Biopsies

Five participants consented to have a biopsy on each leg before the application of the randomized topicals and post procedure at 2 and 4 weeks. Three participants’ biopsies were sent for gene analysis, and 2 participants’ biopsies were sent to an independent dermatopathologist for analysis.

#### Gene Analysis

Significant differential gene expression patterns were observed between the bland moisturizer and the experimental group in comparison to the pretreatment biopsy. Compared with the bland moisturizer, the experimental group demonstrated a hastened immune inflammatory response moving more rapidly to an anti-inflammatory reversal at 2 weeks followed by a wound healing extracellular remodeling effect at 4 weeks. A more detailed analysis of the gene expression changes appears in a paper purely devoted to this gene analysis.^8^ However, to summarize:

### Comparison of the Biopsies Collected at 2 Weeks From the Untreated and Treated Groups

The gene expression analysis of the biopsies collected at 2 weeks from the untreated and treated groups revealed that 15 genes were significantly upregulated and 5 were significantly downregulated in the untreated group compared with the pretreatment group. On the other hand, 25 genes were significantly upregulated and 3 were significantly downregulated in the treated group compared with the pretreatment group.^8^

The upregulated pathways on the comparator side are considered pro-inflammatory regulators. In contrast, the unique pathways enriched in the treated group triggered anti-inflammatory, immunomodulatory, and clearance responses. Taken together, the biopsies collected at 2 weeks from the treated and untreated sides showed strikingly different patterns of gene expression. The untreated side revealed typical inflammatory signaling that would be expected in response to the given treatment. In contrast, the treated side revealed that the inflammatory response favored pro-clearance and preparation for healing.

### Comparison of the Biopsies Collected at 2 and 4 Weeks From the Treated Group

The gene expression data from the biopsy samples collected from the 4-week untreated group revealed that only 2 genes (*CHRNA7* and *IL-11*) were differentially upregulated in comparison to the pretreatment sample. These results indicated that by 4 weeks, the untreated group had essentially returned to baseline. However, in the 4-week treated group, 18 genes were significantly upregulated compared with the pretreatment sample. The 4 unique pathways enriched in the 4-week group were related not only to anti-inflammatory signaling but also to macrophage regulation and ECM remodeling. Together, these data provide evidence that the treatment initially activated the immune system to stimulate clearance and protect the tissue from harm. As time went on, the treatment abolished the pro-inflammatory signature and stabilized an anti-inflammatory environment for the promotion of new ECM and further healing.^8^

#### Histological Analysis

**Figure 6.**
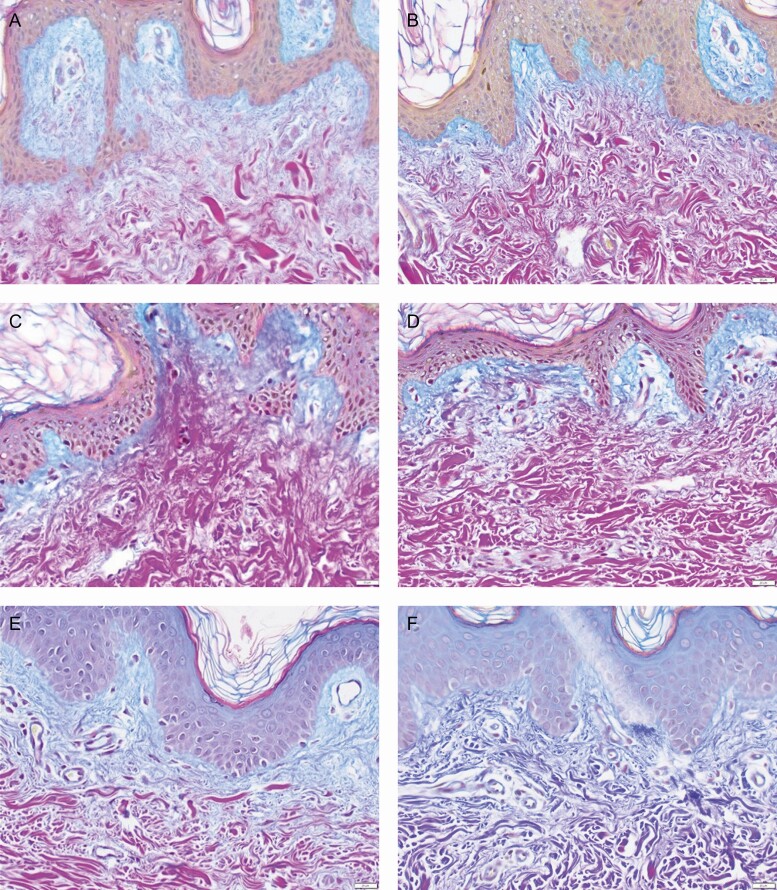
Herovici Stain 40× Subject 4 comparator side and RSN/TFB side at baseline, 2 weeks, and 4 weeks. Note the gradual and then dramatic shift at 4 weeks of laying down of early collagen fiber (mucopolysaccharides) in the papillary dermis on the RSN/TFB side represented by conversion from magenta color to blue color. (a) Right pretreatment, (b) Left pretreatment, (c) Bland moisturizer—2 weeks post procedure, (d) RSN/TFB—2 weeks post procedure, (e) Bland moisturizer—4 weeks post procedure, and (f) RSN/TFB—4 weeks post procedure. TFB, TransFORM Body Treatment with TriHex Technology; RSN, Regenerating Skin Nectar with TriHex Technology.

The Herovici stain has been particularly useful to demonstrate new collagen formation by staining newly laid down mucopolysaccharide components in the papillary dermis denoting early collagen regeneration. This serves to establish the regenerative changes in the ECM that would be sought with products initiating antiaging changes in the skin. Analysis of the biopsies from the dermatopathologist shows a marked increase in neocollagenesis particularly on the RSN/TFB side (magenta to blue conversion). In addition, increased fibrillin, elastin component, was consistently elevated at 4 weeks in both participants on the RSN/TFB side (Figure 6).

**Figure 7.**
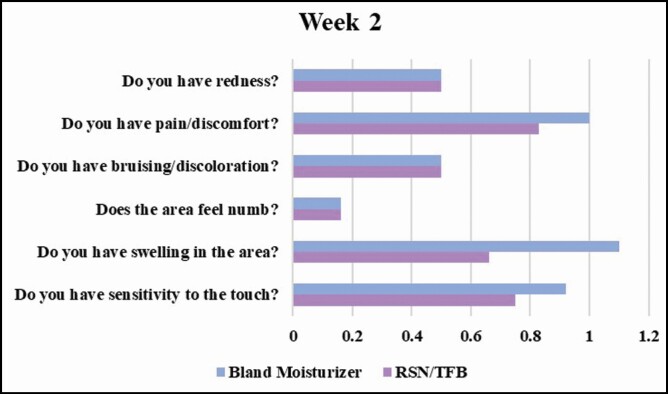
Mean participant assessments—week 2.

As far as the participant assessments are concerned, in keeping with signs and symptoms being maximal within the first 2 weeks, swelling on the topical treatment side was assessed as less severe by the majority of participants at week 2, mean average score for swelling on the topical treatment side .66 and 1.1 on the bland moisturizer leg. It is also of note that 5 of the 6 participants selected to use the topical treatments in their next body procedure (Figure 7).

**Figure 8.**
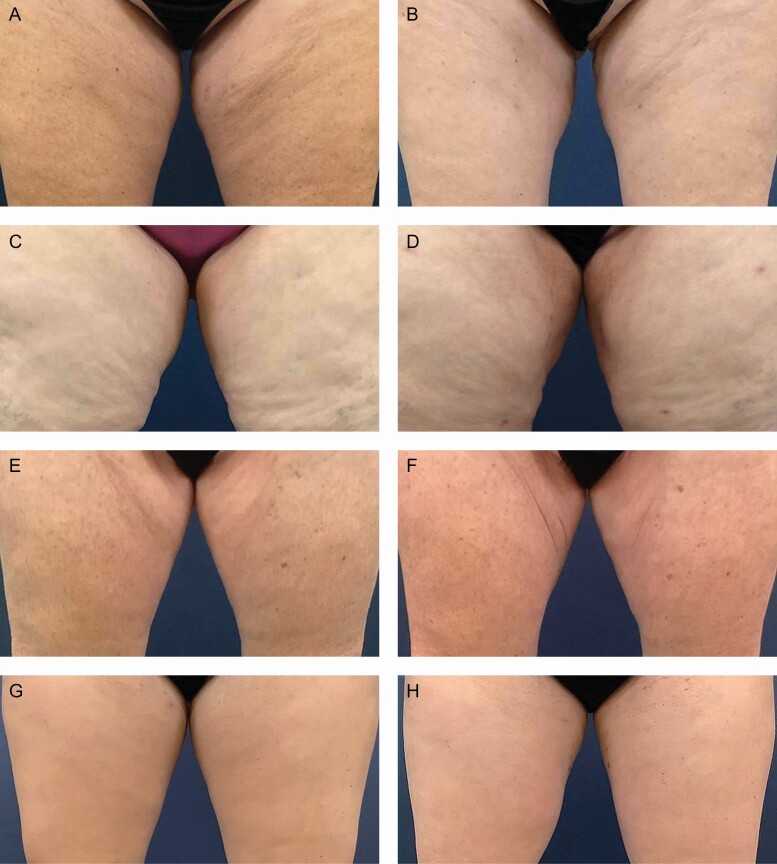
Representative photographs of 4 female participants at pretreatment, 10 weeks post follow-up showing improvement in skin tone and reduced irregularities in the area of medial thigh liposuction on the RSN/TFB side. Female participant 1, age 48 (a) pretreatment and (b) 10 weeks post procedure-treated left side with RSN/TFB. Female participant 2, age 43 (c) pretreatment and (d) 10 weeks post procedure-treated right side with RSN/TFB. Female participant 3, age 28 (e) pretreatment and (f) 10 weeks post procedure-treated left side with RSN/TFB. Female participant 4, age 58 (g) pretreatment and (h) 10 weeks post procedure-treated left side with RSN/TFB. TFB, TransFORM Body Treatment with TriHex Technology; RSN, Regenerating Skin Nectar with TriHex Technology.

**Figure 9.**
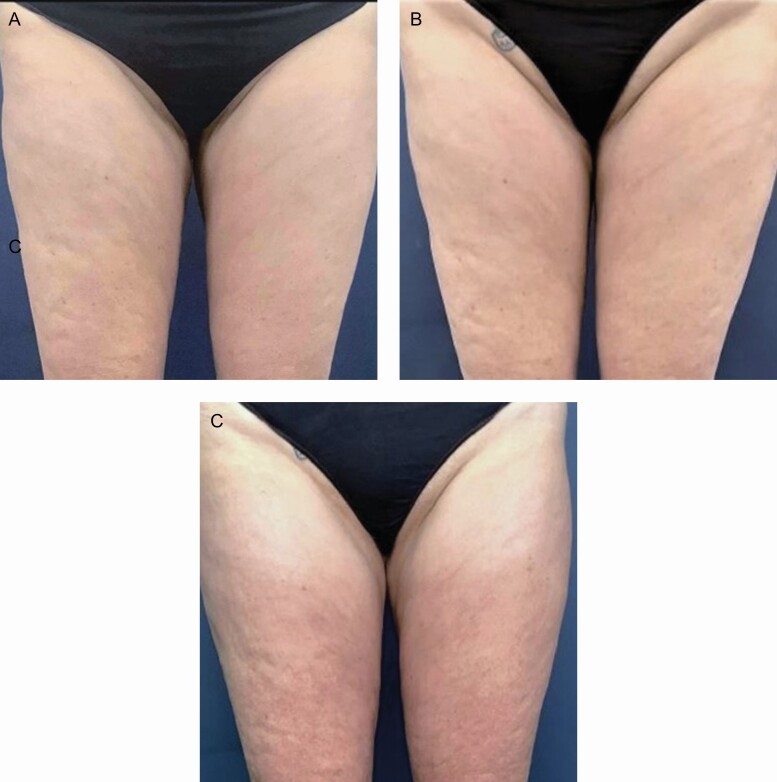
Representative photographs of a female participant age 53 at pretreatment (a) 10 weeks post follow-up, (b) at 10 months post follow-up, and (c) long-term follow-up demonstrating consistent improvement of skin tone. RSN/TFB-treated left side. TFB, TransFORM Body Treatment with TriHex Technology; RSN, Regenerating Skin Nectar with TriHex Technology.

**Figure 10.**
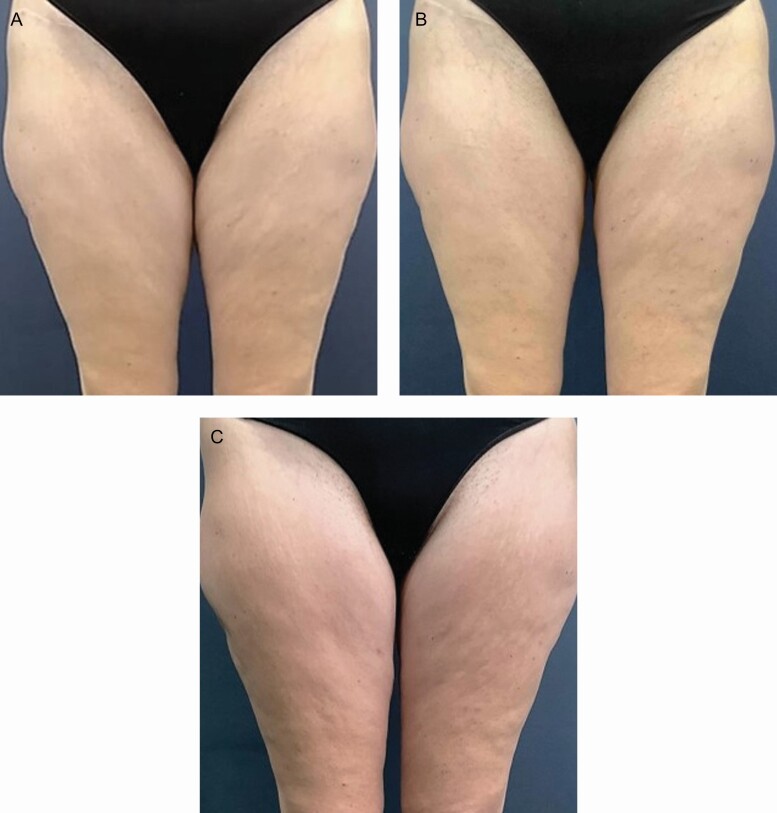
Representative photographs of a female participant age 46 at pretreatment (a) 10 weeks post follow-up, (b) at 11 months post follow-up, and (c) long-term follow-up demonstrating consistent improvement of skin tone. RSN/TFB-treated right side. TFB, TransFORM Body Treatment with TriHex Technology; RSN, Regenerating Skin Nectar with TriHex Technology.

Representative participant photographs are available as Figures 8-10. 

## DISCUSSION

As we age and as the skin is exposed to the sun and other environmental factors, it leads to a compromised ECM. For a procedure to have an optimal result, it is essential to clear out the ECM of the damaged debris and make room for new collagen and elastin to support the recovery and outcomes. This damaged debris is also relevant to surgical procedures involving fat tissue, where excessive manipulation from liposuction cannulae, Bovie, or even pure surgical resection of fat tissue results in the release of lipid droplets (triglycerides and free fatty acids) from the damaged fat cell. These lipid droplets are inflammatory in nature and cause localized reactions of swelling, induration, and, in extreme cases, fat necrosis. These lipid droplets are large and cannot easily be digested by macrophages. These large particles, which are released by adipocytes, are digested through autophagy (lipophagy), which alters these large lipid droplets into smaller products that can be taken up by macrophages, thus providing clearance.^4,9,10^ Respectively, RSN and TFB have been used pre and post procedure and have demonstrated hastened healing, improved outcomes, and acceleration of autophagy.^2-7^ In this study, the preprocedural skin preparation combined with the postprocedural treatment further confirmed the improved healing seen by the physician and participants and correlated to the objective measurements and gene analysis results. On the side using RSN and TFB, induration and edema along with subcutaneous fibrous banding were less severe from weeks 1–4 and 10. This followed on trend with the induration skinfibrometer results that had a lower change from baseline at weeks 1–2 when you would typically see the most induration. Most importantly, gene expression studies confirmed a hastened inflammatory phase converting more rapidly to the anti-inflammatory regenerative healing milieu with evidence of extracellular remodeling only present on the RSN/TFB topical side at week 4 and histological biopsies demonstrated improved collagenesis and elastogenesis on the treated side as well.

Limitations to this study include a small sample size; however, triple replicates were conducted for gene analysis, biopsies, and fibrometer readings increasing data size considerably. Even with the small sample size, gene ontology showed dramatic differences in all patients between the 2 sides.

## CONCLUSIONS

Using RSN to prepare the skin for surgical procedures combined with RSN and TFB post procedure has been correlated through clinical study outcomes, histological evidence, and finally gene analysis demonstrating remodeling of the ECM, accelerating healing, and initiation of anti-inflammatory genes.^8^ The investigator assessments, participant assessments, and induration measurements align with less severe postprocedural events and match up with the genes being expressed within the 4 weeks postprocedure period.

## Supplementary Material

ojaa055_suppl_Supplementary_Appendix
