# Supplementary material for: Evaluating the Efficacy, Tolerability, and Outcomes of Topical Tripeptide/Hexapeptide Formulations Before and After Liposuction of the Medial Thighs
Source: Aesthet Surg J Open Forum. 2020 Dec 30;3(1):ojaa055. doi: 10.1093/asjof/ojaa055 (PMC7876984; doi:10.1093/asjof/ojaa055)
Supplement: ojaa055_suppl_Supplementary_Appendix [file ojaa055_suppl_supplementary_appendix.docx]

Supplementary Appendix:

**Subject Assessment of Procedure Area (right and left)**

**Please check one box for each question. Thank you.**

|  | | **None (0)** | **Mild (1)** | **Moderate (2)** | **Severe (3)** |
| --- | --- | --- | --- | --- | --- |
| 1 | Do you have sensitivity to the touch? |  |  |  |  |
| 2 | Do you have swelling in the area? |  |  |  |  |
| 3 | Does the area feel numb? |  |  |  |  |
| 4 | Do you have bruising/discoloration? |  |  |  |  |
| 5 | Do you have pain/discomfort? |  |  |  |  |
| 6 | Do you have redness? |  |  |  |  |
| 7 | Does the area feel soft and flexible? |  |  |  |  |

**Comments:**
